# Monocyte-Derived Macrophage Ferroptosis Amplifies Cholangitis in Primary Biliary Cholangitis via a Calpain/ACSL4 Axis

**DOI:** 10.3390/biomedicines14061208

**Published:** 2026-05-27

**Authors:** Tianfu Liu, Yichen Huang, Yizhe Wang, Rui Zhao, Haili Shen

**Affiliations:** 1 Department of Hepatology, The Second Hospital & Clinical Medical School, Lanzhou University, Lanzhou 730030, China; 2 The Second Hospital & Clinical Medical School, Lanzhou University, Lanzhou 730030, China; 3 Department of Respiratory and Critical Care Medicine, The First People Hospital of Lanzhou, Lanzhou 730050, China; 4 Department of Rheumatology, The Second Hospital & Clinical Medical School, Lanzhou University, Lanzhou 730030, China

**Keywords:** primary biliary cholangitis, monocyte-derived macrophages, ferroptosis, ACSL4, calpain

## Abstract

**Background**: Recruitment and activation of monocyte-derived macrophages (MoMFs) sustain cholangitis in primary biliary cholangitis (PBC), but whether MoMFs amplify inflammation through ferroptosis remains unclear. We defined ferroptotic programs in MoMFs and evaluated the calpain/ACSL4 axis as a regulatory and therapeutic node. **Methods**: We analysed a public human liver single-cell RNA sequencing (scRNA-seq) dataset and examined MoMF-associated ACSL4 and 4-hydroxynonenal (4-HNE) signals in CD11b^+^CD68^+^ cells by multiplex immunofluorescence. We used a 2OA–BSA-induced PBC-like mouse model to assess liver injury, inflammation and ferroptosis-related markers and tested Liproxstatin-1 (Lip-1), rosiglitazone (ROSI) or the calpain inhibitor PD150606. Bone marrow-derived macrophages (BMDMs) from control and PBC mice were profiled and challenged with RSL3, with or without Ferrostatin-1 (Fer-1), ROSI or PD150606. **Results**: MoMFs were expanded in PBC livers and showed the strongest induction of ferroptosis signatures, centered on ACSL4, with enhanced inflammatory crosstalk with cholangiocytes. Human PBC tissues showed increased CD11b^+^CD68^+^ cells positive for ACSL4 or 4-HNE. In PBC-like mice, malondialdehyde (MDA) increased and glutathione (GSH) decreased, and macrophages showed greater colocalization with ferroptosis markers; Lip-1, ROSI or PD150606 improved liver biochemistry, reduced inflammation scores and limited macrophage infiltration. PBC-derived BMDMs upregulated ACSL4 and CAPN1/2 and were more sensitive to RSL3; Fer-1, ROSI or PD150606 attenuated ferroptosis-associated molecular changes. **Conclusions**: MoMF ferroptosis is prominently engaged in PBC, and our findings implicate a pharmacologically tractable calpain/ACSL4 axis that may contribute to macrophage ferroptotic susceptibility and inflammatory liver injury.

## 1. Introduction

Primary biliary cholangitis (PBC) is a chronic autoimmune cholestatic liver disease characterized by progressive, non-suppurative destruction of the small intrahepatic bile ducts [1]. The incidence is estimated at 1.8 per 100,000 person-years and the prevalence at 18.1 per 100,000 persons [2]. Over recent decades, the prevalence has increased worldwide, with the most pronounced rise reported in the Western Pacific region [2,3]. Women account for 80–90% of cases, and disease onset most commonly occurs between 40 and 70 years of age [1,4]. Although the aetiology and pathogenesis of PBC remain incompletely understood, current evidence suggests that disease onset reflects an aberrant immune response to environmental triggers in genetically susceptible individuals [5]. Loss of immune tolerance to biliary epithelial cells drives chronic cholangitis and cholestasis, thereby promoting bile acid-mediated inflammation and progressive liver injury that can culminate in biliary cirrhosis and liver failure [6]. No curative therapy is currently available. Ursodeoxycholic acid (UDCA) and obeticholic acid (OCA), the standard pharmacologic treatments, improve transplant-free survival, but therapeutic responses vary substantially among patients.

Macrophages are the most abundant innate immune cells in the liver, comprising approximately 20% of all non-parenchymal cells in the healthy liver, and are essential for immune defense, tissue remodeling, and the maintenance of hepatic homeostasis [7]. Hepatic macrophages comprise resident Kupffer cells, which arise from embryonic yolk sac progenitors, and monocyte-derived macrophages (MoMFs), which originate from circulating monocytes that infiltrate the liver. In patients with PBC, CD14^+^CD16^+^ monocytes, a precursor pool for MoMFs, are markedly increased, and expansion of the CD14^low^CD16^+^ subset correlates with the severity of liver injury and the abundance of Th1 cells, consistent with a pro-inflammatory role [8]. Peripheral monocytes from patients with PBC also show increased ARID3A expression, which suppresses downstream MERTK signalling and impairs macrophage-mediated clearance of apoptotic biliary epithelial cells (BECs); this defect promotes the accumulation of apoptotic cells and exacerbates cholestatic injury [9]. In PBC liver tissue, IBA1^+^ macrophages cluster within CK19^+^ ductular reaction areas and display pronounced phenotypic heterogeneity linked to spatial localization [10]. Data from cholestatic mouse models further indicate that macrophages in these ductular regions are predominantly MoMFs [10]. Moreover, in murine PBC models, CX3CR1^hi^CD11c^+^ MoMFs have been identified as a major source of interleukin-23 (IL-23), thereby driving Th17-axis-mediated inflammation [11]. Collectively, these observations suggest that the biliary microenvironment selectively recruits and reprograms MoMFs, sustaining a feed-forward inflammatory and profibrotic programme that contributes to PBC progression.

Ferroptosis is a regulated form of cell death driven by iron-dependent lipid peroxidation. It is characterized by intracellular accumulation of ferrous iron (Fe^2+^) and lipid peroxides in polyunsaturated fatty acid-containing phospholipids (PUFA-PLs), culminating in loss of plasma-membrane integrity and cell death [12]. Acyl-CoA synthetase long-chain family member 4 (ACSL4) lowers the threshold for ferroptosis by activating polyunsaturated fatty acids (PUFAs) and promoting their incorporation into membrane phospholipids, thereby expanding the pool of peroxidation-prone PUFA-PL substrates; genetic ablation or pharmacological inhibition of ACSL4 markedly reduces cellular susceptibility to ferroptosis [13,14]. In contrast, antioxidant systems such as GPX4/glutathione (GSH) and the cystine/glutamate antiporter system Xc^−^ (SLC7A11/xCT–SLC3A2) limit PUFA-PL peroxidation and increase the ferroptotic threshold [13,14]. Emerging evidence further implicates macrophage ferroptosis in amplifying inflammation in chronic inflammatory and autoimmune disorders [15,16,17]. In this context, calpain has been proposed as an upstream regulator of ACSL4 expression or activity, forming a putative calpain/ACSL4 axis that may govern macrophage ferroptosis [18,19]. However, within the biliary microenvironment of PBC, direct evidence for ferroptosis in MoMFs and its regulation remains lacking.

Here, we integrated human liver single-cell transcriptomics with supportive tissue-level evidence and functional studies in a PBC-like mouse model and primary bone marrow-derived macrophages (BMDMs) to define ferroptotic susceptibility in MoMFs and its contribution to inflammatory amplification. We further interrogated upstream control and therapeutic tractability of the calpain/ACSL4 axis. Together, our findings provide mechanistic and translational evidence linking ferroptosis to PBC pathophysiology and support targeting this pathway as a potential therapeutic strategy.

## 2. Materials and Methods

### 2.1. Single-Cell RNA-Sequencing Data Acquisition and Preprocessing

Raw expression matrices from the GEO dataset GSE243981 (2 patients with PBC and 6 healthy controls) were downloaded and imported into R as Seurat objects (Seurat v5.3). Cell-level quality control excluded cells with <200 or >10,000 detected genes, <1000 unique molecular identifiers (UMIs), or >20% mitochondrial transcripts. Genes detected in fewer than three cells were removed. Doublets were identified and removed using DoubletFinder, with the expected doublet rate set at 5%. The remaining cells were normalized and scaled using the NormalizeData and ScaleData functions, and the top 2000 highly variable genes were identified with FindVariableFeatures [20,21].

### 2.2. Single-Cell RNA-Sequencing Data Analysis

#### 2.2.1. Batch Correction, Dimensionality Reduction, and Clustering

Harmony was applied in principal-component space, and the first 12 principal components were retained for downstream analyses [22]. Louvain clustering based on a shared nearest-neighbor graph was used to identify 9 cell clusters. Two-dimensional visualization was performed using UMAP, and differentially expressed genes (DEGs) for each cluster were identified with the FindAllMarkers function. Cell types were annotated on the basis of previously reported canonical marker genes together with cluster-specific DEGs identified in this study [23,24].

#### 2.2.2. Ro/e Analysis of Cellular Composition

To compare cell-type composition between conditions, we calculated, for each cell cluster, the ratio of observed to expected cell counts (Ro/e). Clusters with Ro/e > 1 were considered enriched in the disease group, whereas clusters with Ro/e < 1 were considered relatively depleted in the disease group [25].

#### 2.2.3. Differential Expression Analysis in MoMF Subsets

MoMFs were extracted from the integrated object based on cell-type annotations. For each sample, raw UMI counts within the MoMF subset were summed by gene to generate a pseudo-bulk count matrix (genes × samples). Differential expression was performed using DESeq2, with log_2_ fold-change (log_2_FC) shrinkage applied using apeglm to improve effect-size stability [26]. Wald test *p* values were adjusted for multiple testing using the Benjamini–Hochberg procedure; genes with |log_2_FC| ≥ 1 and FDR < 0.05 were considered differentially expressed. Differential expression results were visualized as specified in the corresponding figure legends.

#### 2.2.4. Pathway Enrichment Analysis

Gene set enrichment analysis (GSEA) was performed on the full gene list ranked by the Wald statistic using clusterProfiler [27]. Ferroptosis-related pathways were assessed using FerrDb V2 gene sets (http://www.zhounan.org/ferrdb, accessed on 17 October 2024), and additional pathway gene sets were obtained from KEGG [28]. Over-representation analysis (ORA) of KEGG pathways and Gene Ontology (GO) terms was conducted separately for upregulated and downregulated differentially expressed genes [29,30]. GSEA results were reported as normalized enrichment scores (NES) and false-discovery-rate (FDR) q values, whereas ORA results are summarized by GeneRatio and adjusted *p* values (Benjamini–Hochberg).

#### 2.2.5. Ferroptosis Activity Scoring

Using FerrDb V2 ferroptosis gene sets, a ferroptosis module score (FerrScore) was computed for each cell with AddModuleScore [28,31]. Scores were compared between PBC and control samples using two-sided Wilcoxon rank-sum tests, with multiple testing controlled by Benjamini–Hochberg adjustment where applicable. Within MoMFs, associations between FerrScore and candidate gene expression were assessed using two-sided Spearman correlation tests.

#### 2.2.6. Cell–Cell Communication Analysis

Intercellular communication was inferred using CellChat (v2.1.0), which predicts signaling interactions based on ligand–receptor pairs and cofactors [32]. Separate CellChat objects were generated for the control and disease groups and then merged for comparative analysis to identify cell clusters with significantly altered signaling activity and to highlight key ligand–receptor pairs and signaling pathways.

#### 2.2.7. Single-Cell Trajectory and Pseudotime Analysis

Single-cell trajectories of MoMF subsets from control and disease samples were reconstructed using Monocle2 [33]. In Monocle2, pseudotemporal trajectories were inferred and pseudotime values were obtained with the reduceDimension function. Trajectories and dynamic gene-expression patterns were visualized with the plot_cell_trajectory and plot_pseudotime_heatmap functions. Along the pseudotime axis, cells were divided into “Low” and “High” groups according to the median pseudotime, and differences in module (gene-set) scores between these groups were assessed.

### 2.3. Human Liver Specimens

Liver tissue samples were obtained from 3 patients with PBC and 3 individuals with normal liver tissue undergoing surgical resection of hepatic hemangioma. The diagnosis of PBC was established according to accepted criteria. Patients fulfilled ≥2 of the following three criteria: (1) biochemical evidence of cholestasis (predominantly elevated alkaline phosphatase (ALP) and/or γ-glutamyltransferase (GGT)), with imaging excluding biliary obstruction and large-duct disease; (2) positivity for antimitochondrial antibodies (AMA), including the AMA-M2 subtype; and (3) histological evidence of non-suppurative destructive cholangitis with injury to small intrahepatic bile ducts. Written informed consent was obtained from all participants prior to tissue collection. The study protocol was in accordance with the Declaration of Helsinki and was approved by the Ethics Committee of the Second Hospital of Lanzhou University (Approval No. 2025A-1253).

### 2.4. Animal Model

Female C57BL/6J mice (6–8 weeks old; 18–22 g) were obtained from the Laboratory Animal Center of the Lanzhou Veterinary Research Institute, Chinese Academy of Agricultural Sciences. Mice were housed under controlled conditions (20–22 °C, 50–60% humidity) with a 12 h light/12 h dark cycle and provided food and water ad libitum. All procedures were approved by the Institutional Animal Care and Use Committee of the Lanzhou Veterinary Research Institute, Chinese Academy of Agricultural Sciences, and conducted in accordance with the National Institutes of Health Guide for the Care and Use of Laboratory Animals.

The 2-octynoic acid-bovine serum albumin (2OA-BSA) conjugate was synthesized as previously described [34]. To induce a PBC-like model, 100 μg 2OA-BSA was dissolved in 50 μL sterile phosphate-buffered saline (PBS) and emulsified with 50 μL complete Freund’s adjuvant (CFA) (total volume, 100 μL). Mice received an intraperitoneal injection of the 2OA-BSA/CFA emulsion on day 0, followed by booster immunizations on days 15 and 29 with 2OA-BSA emulsified in incomplete Freund’s adjuvant (IFA) using the same dose and volume [35]. Reagents and compounds are listed in Table 1.

### 2.5. Animal Grouping and Treatment

Healthy control mice received phosphate-buffered saline (PBS) by intraperitoneal (i.p.) injection (*n* = 6). PBC-induced mice were randomly assigned to four groups (*n* = 6 per group) with comparable baseline body weights: (1) PBC model group (vehicle control, PBS); (2) PBC + Liproxstatin-1 (Lip-1; 10 mg/kg/day, i.p.) from day 30 for 28 consecutive days; (3) PBC + rosiglitazone (ROSI; 0.5 mg/kg/day, i.p.) from day 30 for 28 consecutive days; and (4) PBC + PD150606 (3 mg/kg/day, i.p.) from day 30 for 28 consecutive days. Lip-1, ROSI and PD150606 were dissolved in dimethyl sulfoxide (DMSO) and diluted in PBS immediately before administration (100 μL per injection). At the end of the treatment period, mice were euthanized by cervical dislocation performed by trained personnel in accordance with the approved animal protocol, and blood, liver, and bone marrow were collected for downstream analyses.

### 2.6. Isolation, Culture, and Treatment of BMDMs

BMDMs were generated as described previously [35]. Briefly, femurs and tibias were harvested aseptically from control and PBC model mice, and bone marrow cells were flushed, washed and subjected to red blood cell lysis. Cells were then cultured for 7 days in DMEM supplemented with macrophage colony-stimulating factor (M-CSF; 10 ng/mL) to obtain mature BMDMs. Ferroptosis was induced by RSL3 (0.5 μM) for 5 h. For rescue or intervention experiments, cells were pretreated with Ferrostatin-1 (1 μM), rosiglitazone (5 μM), or PD150606 (20 μM) for 1 h, followed by RSL3 (0.5 μM) for 5 h. All cells were maintained at 37 °C in a humidified incubator with 5% CO_2_.

### 2.7. Bulk RNA Sequencing of BMDMs

Total RNA was extracted from mature BMDMs using TRIzol (R401-01, Vazyme, Nanjing, China) according to the manufacturer’s instructions. Poly(A)+ RNA was used for strand-specific library preparation, and libraries were sequenced in paired-end mode on an Illumina NovaSeq 6000 platform (OE Biotech, Shanghai, China). After quality control, reads were aligned to the mouse reference genome and gene expression was quantified. Differential expression analysis (DESeq2) and pathway analyses (GSEA and KEGG/GO over-representation analysis using clusterProfiler) were performed as described above.

### 2.8. Liver Function Tests and Antimitochondrial Antibody Levels

Serum alanine aminotransferase (ALT), aspartate aminotransferase (AST) and alkaline phosphatase (ALP) were measured using an automated biochemical analyzer (BS-360E, Mindray, Shenzhen, China). Serum antimitochondrial antibody M2 (AMA-M2) levels were quantified using a mouse AMA-M2 ELISA kit (ml037676, Mlbio, Shanghai, China) according to the manufacturer’s instructions.

### 2.9. Histological Staining

Liver tissues were fixed in 4% paraformaldehyde (pH 7.2), dehydrated through a graded ethanol series, cleared in xylene, and embedded in paraffin. Sections were cut at a thickness of 4 μm and stained with hematoxylin and eosin (H&E). Inflammatory activity and stage were evaluated according to the modified histologic activity index (mHAI) [36]. All slides were independently evaluated in a blinded manner by two investigators, and any discrepancies were resolved by joint review until consensus was reached.

### 2.10. Immunohistochemical Staining

Paraffin-embedded mouse liver sections were deparaffinized, rehydrated, and subjected to heat-induced antigen retrieval. Endogenous peroxidase activity was quenched with 3% H_2_O_2_, followed by blocking with PBS containing 10% bovine serum albumin (BSA) at room temperature for at least 30 min. Sections were then incubated overnight at 4 °C with primary antibodies against F4/80 (28463-1-AP, Proteintech, Wuhan, China), ACSL4 (22401-1-AP, Proteintech, China), GPX4 (67763-1-Ig, Proteintech, China), or 4-HNE (bs-6313R, Bioss, Beijin, China). Species-matched horseradish peroxidase (HRP)-conjugated secondary antibodies were applied for 1 h at room temperature. Signals were developed using 3,3′-diaminobenzidine (DAB), and nuclei were counterstained with hematoxylin. Sections were mounted with neutral resin and imaged under identical acquisition settings for quantitative analysis.

### 2.11. Immunofluorescence Staining

Paraffin-embedded human and mouse liver sections were deparaffinized, rehydrated, subjected to antigen retrieval, and blocked with serum. Multiplex staining was performed according to host species and fluorophore compatibility. For human liver tissue, CD68 (PA5-109344, Invitrogen, Carlsbad, CA, USA) and CD11b (14-0112-82, Invitrogen, USA) were co-stained with ACSL4 (22401-1-AP, Proteintech, China) or 4-HNE (bs-6313R, Bioss, China). For mouse liver tissue, F4/80 (28463-1-AP, Proteintech, China) was co-stained with ACSL4 or 4-HNE. Sections were incubated with primary antibodies overnight at 4 °C, followed by species-matched fluorescent secondary antibodies the next day in the dark. Nuclei were counterstained with DAPI, and slides were mounted with an antifade medium. Image acquisition settings were kept constant between groups.

### 2.12. Western Blotting

Liver tissues and cultured cells were lysed on ice in universal lysis buffer containing protease inhibitors (AIWB-012, Affinibody, Wuhan, China). Lysates were centrifuged to remove debris, and the supernatant was collected as total protein. Protein concentrations were determined with a BCA assay kit (P0010, Beyotime, Shanghai, China). Depending on the molecular weight of the target proteins, samples were separated on 10% or 12% SDS-polyacrylamide gels (SDS-PAGE) and transferred to polyvinylidene fluoride (PVDF) membranes. Membranes were blocked for 10 min at room temperature with Minute Block low-background blocking buffer (AIWB-004, Affinibody, China). Membranes were then incubated overnight at 4 °C with primary antibodies diluted according to the manufacturer’s instructions: CAPN1 (10538-1-AP), CAPN2 (11472-1-AP), ACSL4 (22401-1-AP), GPX4 (67763-1-Ig), SLC7A11 (26864-1-AP), and FTH1 (11682-1-AP), all from Proteintech (Wuhan, China). After washing with TBST, membranes were incubated with appropriate HRP-conjugated secondary antibodies for 1 h at room temperature. Signals were detected with a high-sensitivity ECL chemiluminescence kit (AIWB-006, Affinibody, China), and band intensities were quantified using ImageJ software version 1.53t.

### 2.13. Measurement of Malondialdehyde and Glutathione

Malondialdehyde (MDA) and total glutathione (GSH) were quantified in tissue and cell lysates according to the manufacturer’s protocols. MDA levels were measured using an MDA assay kit (S0131S, Beyotime, China). Total GSH was measured using a GSH assay kit (S0053, Beyotime, China). Results were expressed as μmol per gram of tissue for liver samples and as nmol per milligram of protein for cell samples.

### 2.14. Cell Viability Assay

Cell viability was assessed using a CCK-8 kit (C0041, Beyotime, China) according to the manufacturer’s instructions. Absorbance at 450 nm was measured, and relative cell viability was calculated from the optical density values.

### 2.15. Statistical Analysis

Unless otherwise stated, data are presented as the mean ± standard deviation (SD). Statistical analyses were performed with GraphPad Prism 10 (GraphPad Software, Boston, MA, USA). Two-group comparisons were conducted using two-tailed unpaired Student’s *t*-tests; when assumptions were not met, the Mann–Whitney U test was used. Comparisons among multiple groups were performed using one-way ANOVA followed by Tukey’s post hoc test; when ANOVA assumptions were not met, the Kruskal–Wallis test followed by Dunn’s post hoc test was applied. *p* < 0.05 was considered statistically significant.

## 3. Results

### 3.1. Single-Cell Analysis Reveals MoMF Enrichment in PBC Livers

**Figure 1:**
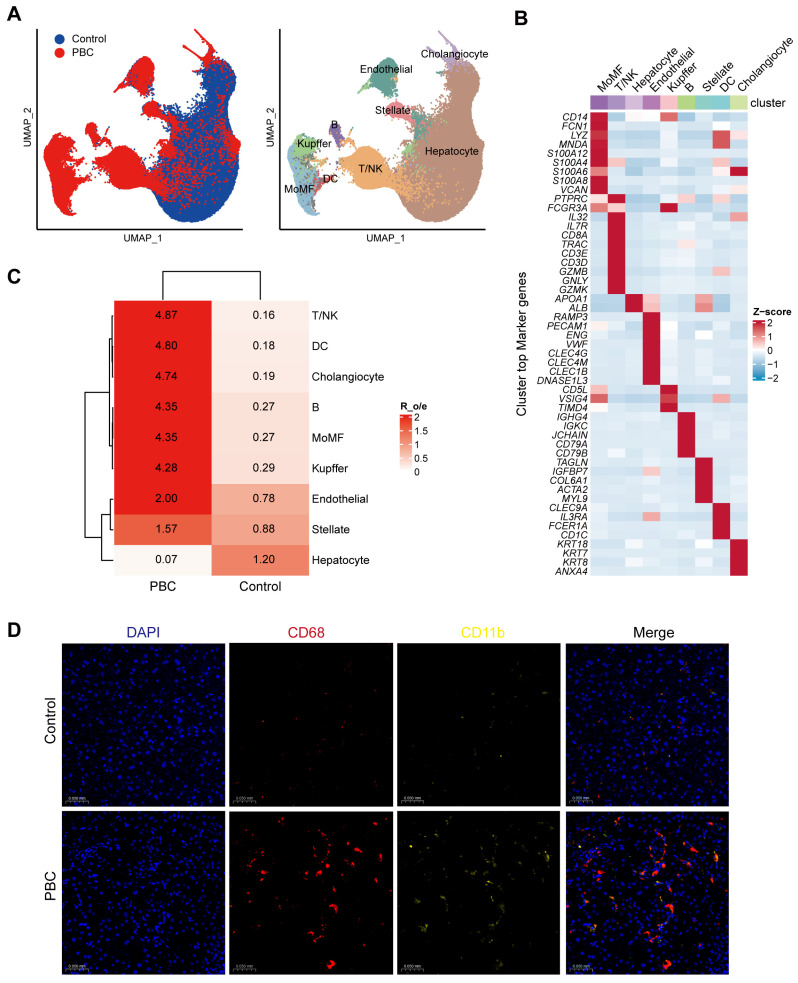
Single-cell and histologic evidence of MoMF enrichment in PBC livers. (**A**) Integrated single-cell UMAP, colored by disease status (control vs. PBC; left) and annotated cell type (right). (**B**) Heatmap showing expression of representative marker genes across the major cell types. (**C**) Heatmap of sample-level cell-type composition, showing the relative abundance of major lineages and a marked increase in monocyte-derived macrophages (MoMFs) in PBC livers. (**D**) Representative multiplex immunofluorescence images of portal tracts from control and PBC liver specimens stained for CD11b (yellow), CD68 (red) and DAPI (blue).

To define changes in hepatic cellular composition in PBC, we analysed integrated single-cell transcriptomic data from liver tissue of patients with PBC and healthy controls. UMAP of the combined dataset captured major parenchymal and immune compartments in both groups, while suggesting altered immune-cell representation in PBC (Figure 1A). Following dimensionality reduction and unsupervised clustering, we annotated nine major lineages based on canonical marker genes: hepatocytes, cholangiocytes, endothelial cells, stellate cells, T/NK cells, B cells, MoMFs, Kupffer cells and dendritic cells (DC). Cluster-specific marker profiles were consistent with reported signatures, supporting the accuracy of cell-type annotation (Figure 1B). Quantification of lineage proportions at the sample level revealed a marked compositional shift in PBC livers, with a prominent increase in MoMFs relative to controls (Figure 1C). To corroborate these findings, we performed dual immunofluorescence staining in an independent set of human liver specimens. Consistent with the single-cell results, CD11b^+^CD68^+^ macrophages were enriched and more densely distributed within and around portal tracts in PBC livers compared with controls (Figure 1D), supporting increased infiltrating macrophages and MoMF enrichment in PBC.

### 3.2. MoMFs Undergo Proinflammatory Reprogramming and Show Enhanced Crosstalk with Cholangiocytes in PBC

**Figure 2:**
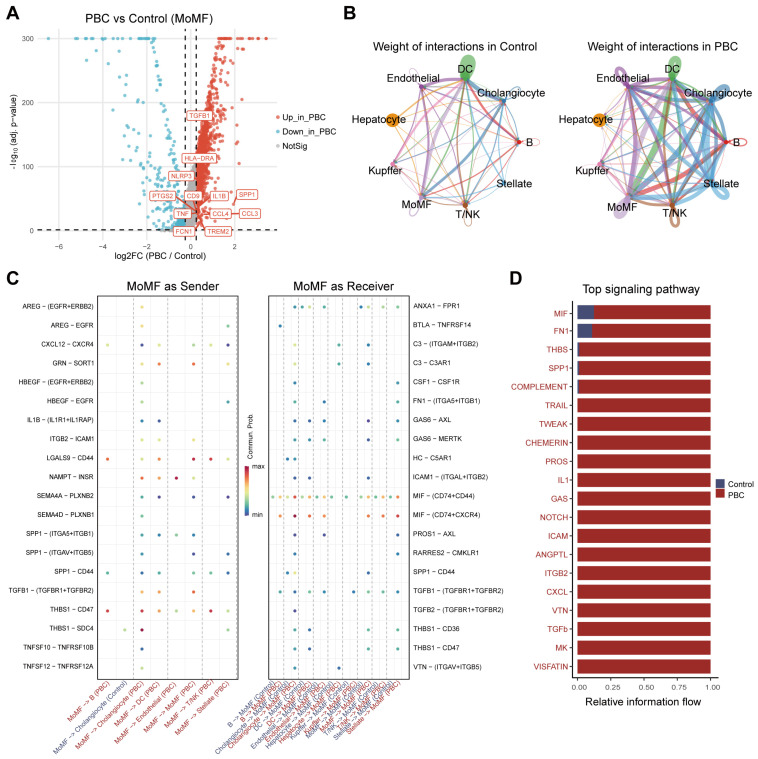
MoMFs undergo inflammatory transcriptional reprogramming and show enhanced crosstalk with cholangiocytes in PBC. (**A**) Volcano plot of differentially expressed genes in MoMFs from PBC versus control livers; red dots indicate upregulated genes and blue dots indicate downregulated genes in PBC, with selected inflammation-related genes labelled. (**B**) Chord diagrams depicting predicted cell–cell communication among major hepatic lineages in control and PBC groups. (**C**) Bubble plots of representative ligand–receptor interactions in which MoMFs act as signal senders or receivers; bubble size indicates communication probability and color indicates interaction strength. (**D**) Bar plot showing relative information flow of the top signaling pathways in the inferred communication network.

Given the marked expansion of MoMFs in PBC livers and their reported contribution to disease immunopathology, we next examined MoMF transcriptional programs and intercellular communication. Differential expression analysis revealed significant induction of inflammatory and innate immune genes in PBC-associated MoMFs, including IL1B, TNF, NLRP3, HLA-DRA and TGFB1, consistent with an inflammatory activation state (Figure 2A). We then assessed ligand–receptor communication to determine how MoMF activation reshapes the hepatic signaling network. Global interaction analysis demonstrated a marked increase in predicted signaling between MoMFs and cholangiocytes in PBC, alongside enhanced interactions with hepatic stellate cells and multiple immune and endothelial populations (Figure 2B). At the level of individual ligand–receptor pairs, MoMFs showed stronger outgoing inflammatory and adhesion/remodeling signals—such as IL1B-IL1R1/IL1RAP, TNFSF10 (TRAIL)-TNFRSF10B and TGFB1-TGFBR1/2—towards cholangiocytes and other target cells. Conversely, MoMFs exhibited increased incoming signaling through pathways including MIF-CD74-CXCR4/CD44, GAS6/PROS1-MERTK/AXL and ICAM1-ITGAL-ITGB2 (Figure 2C). Pathway-level profiling further indicated that the MoMF-centered communication network in PBC was enriched for inflammatory and innate immune programs, including MIF, SPP1, THBS, complement, IL-1 and TGF-β signaling (Figure 2D). Together, these data suggest that PBC is associated with inflammatory activation of MoMFs and enhanced MoMF–cholangiocyte crosstalk that may contribute to sustained cholangitis-associated signaling.

### 3.3. ACSL4-Centered Ferroptotic Signaling Is Most Prominently Enhanced in PBC MoMFs

**Figure 3:**
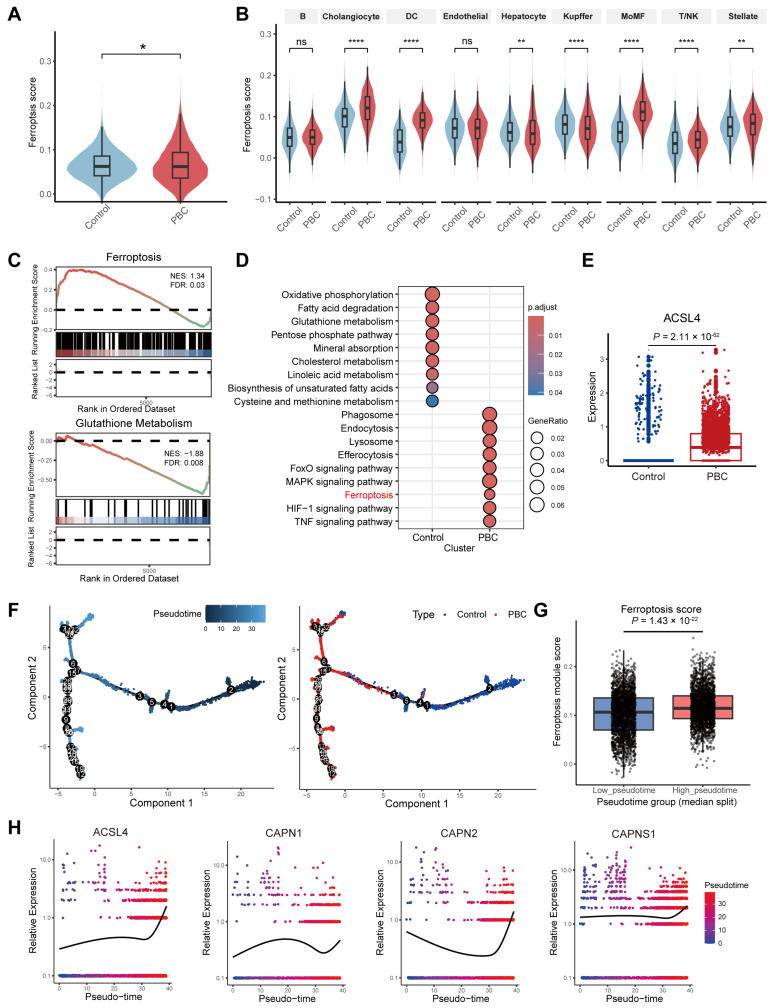
Single-cell transcriptomics reveal ACSL4-centered ferroptosis signatures in MoMFs in PBC. (**A**) Ferroptosis module scores (FerrScore) across all cells in control and PBC livers. (**B**) FerrScore across major cell types in control and PBC groups. (**C**) GSEA of pseudo-bulk MoMF profiles aggregated by sample. (**D**) KEGG pathway over-representation analysis (ORA) of differentially expressed genes in MoMFs, shown as a bubble plot. (**E**) ACSL4 expression in MoMFs in control and PBC livers. (**F**) MoMF pseudotime trajectory colored by pseudotime (left) and by group (control vs. PBC; right). (**G**) FerrScore in MoMFs stratified by the median pseudotime, showing increased scores at later pseudotime. (**H**) Smoothed expression trends of ACSL4, CAPN1, CAPN2 and CAPNS1 along pseudotime (black lines). ns, not significant; * *p* < 0.05; ** *p* < 0.01; **** *p* < 0.0001.

Using FerrDb V2 gene sets, we computed a ferroptosis module score (FerrScore) across the liver single-cell atlas. FerrScore was elevated in PBC relative to controls (Figure 3A), with increases observed across multiple lineages and the largest shift in MoMFs (Figure 3B). To evaluate ferroptosis-related programs at the pathway level, we generated MoMF pseudo-bulk profiles per sample and performed enrichment analyses. GSEA revealed significant positive enrichment of the FerrDb V2 ferroptosis gene set in PBC MoMFs, whereas the KEGG “Glutathione metabolism” gene set was negatively enriched (Figure 3C). Consistently, KEGG over-representation analysis indicated that control MoMFs were enriched for energy metabolism and antioxidant pathways, whereas PBC MoMFs were enriched for ferroptosis and inflammatory signaling pathways (Figure 3D), supporting a shift towards a pro-ferroptotic transcriptional state.

At the gene level, ferroptosis-related transcripts in MoMFs showed upregulation of pro-oxidant and iron-handling genes (including ACSL4, ALOX5, TFRC, HMOX1 and CYBB) with concomitant downregulation of protective genes, including members of the GPX family (Figure S1A); among these, ACSL4 exhibited the most pronounced change (Figure 3E). Calpain components (CAPN1, CAPN2 and CAPNS1) also showed higher detection rates in PBC MoMFs (Figure S1B), and their expression correlated positively with FerrScore and with ACSL4 (Figure S1C), consistent with a calpain-ACSL4 module associated with the ferroptotic signature. We next aligned MoMFs along a pseudotime continuum spanning control and PBC states. FerrScore increased towards later pseudotime (Figure 3F,G), accompanied by progressive induction of ACSL4, CAPN1, CAPN2 and CAPNS1 (Figure 3H), in agreement with the enrichment and correlation analyses.

**Figure 4:**
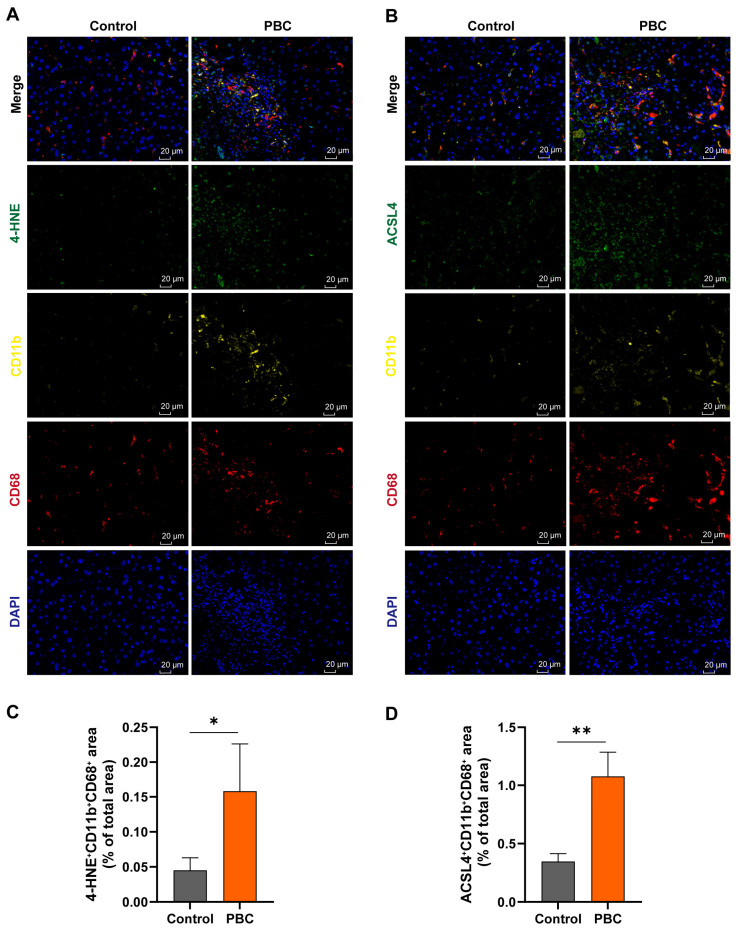
Immunofluorescence evidence of ferroptosis-related markers in infiltrating hepatic macrophages in PBC. (**A**) Representative multiplex immunofluorescence images of portal tracts from control and PBC livers stained for 4-HNE (green), CD11b (yellow), CD68 (red) and DAPI (blue). (**B**) Representative images from the same cohort stained for ACSL4 (green), CD11b (yellow), CD68 (red) and DAPI (blue). (**C**) Quantification of the fractional area of 4-HNE^+^CD11b^+^CD68^+^ colocalization within and around portal tracts. (**D**) Quantification of the fractional area of ACSL4^+^CD11b^+^CD68^+^ colocalization within and around portal tracts. Data are mean ± SD (*n* = 3). * *p* < 0.05, ** *p* < 0.01.

To validate these findings in human tissue, we performed triple immunofluorescence staining for CD11b, CD68 and ACSL4 or 4-HNE in an independent cohort. The fractional colocalized area of CD11b^+^CD68^+^ACSL4^+^ and CD11b^+^CD68^+^4-HNE^+^ signals within and around portal tracts was significantly increased in PBC compared with controls (Figure 4A–D), supporting enhanced lipid peroxidation and ACSL4-linked ferroptotic signaling in infiltrating macrophages and consistent with MoMF enrichment inferred from scRNA-seq.

### 3.4. Hepatic Macrophages in 2OA-BSA-Induced PBC Mice Exhibit a Ferroptosis-Associated Phenotype

**Figure 5:**
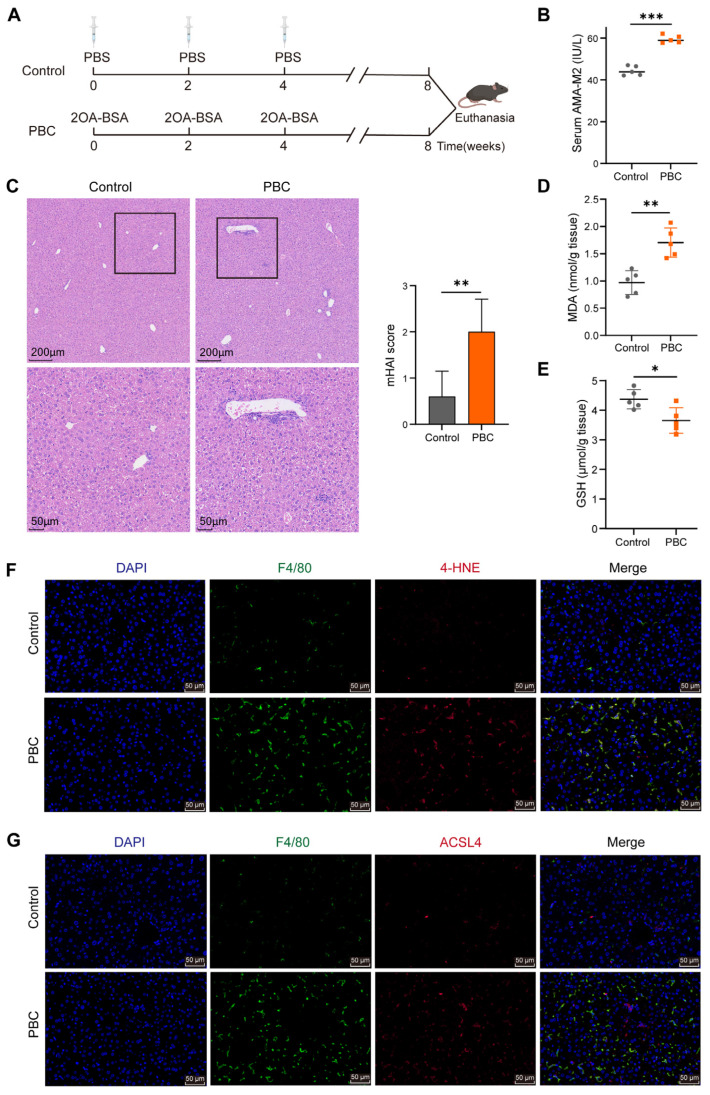
Hepatic macrophages display ferroptosis-associated features in 2OA-BSA-induced PBC-like mice. (**A**) Schematic of the 2OA-BSA immunization protocol used to induce PBC-like cholangitis in mice. (**B**) Serum AMA-M2 levels in control and PBC groups (*n* = 5). (**C**) Representative hematoxylin and eosin (H&E)-stained liver sections and corresponding modified histologic activity index (mHAI) scores (*n* = 5). (**D**,**E**) Hepatic malondialdehyde (MDA) and glutathione (GSH) levels (*n* = 5). (**F**) Representative immunofluorescence images showing F4/80 (green), 4-hydroxynonenal (4-HNE; red) and DAPI (blue). (**G**) Representative immunofluorescence images showing F4/80 (green), ACSL4 (red) and DAPI (blue). Data are mean ± SD. * *p* < 0.05, ** *p* < 0.01, *** *p* < 0.001.

To model PBC-like cholangitis in vivo and examine ferroptosis-associated features in hepatic macrophages, we induced PBC-like disease using 2OA-BSA immunization and euthanized mice after the third scheduled intraperitoneal injection (Figure 5A). Serum AMA-M2 levels were markedly increased in immunized mice relative to controls, consistent with induction of a PBC-related autoantibody response (Figure 5B). Histological assessment revealed dense mononuclear infiltrates in portal tracts with small bile duct injury and architectural distortion, accompanied by significantly higher mHAI scores (Figure 5C). Biochemically, PBC mice exhibited elevated hepatic MDA and reduced GSH levels (Figure 5D,E), indicating enhanced lipid peroxidation and compromised glutathione-dependent antioxidant capacity. Consistent with these redox changes, immunofluorescence showed increased colocalization of F4/80^+^ macrophages with 4-HNE or ACSL4 in PBC livers compared with controls (Figure 5F,G), supporting heightened lipid peroxidation and ACSL4-linked ferroptosis-associated susceptibility in hepatic macrophages. Together, these data establish a PBC-like in vivo context in which hepatic macrophages display ferroptosis-associated features.

### 3.5. Inhibition of Ferroptosis or Calpain/ACSL4 Signaling Attenuates PBC-like Liver Injury

**Figure 6:**
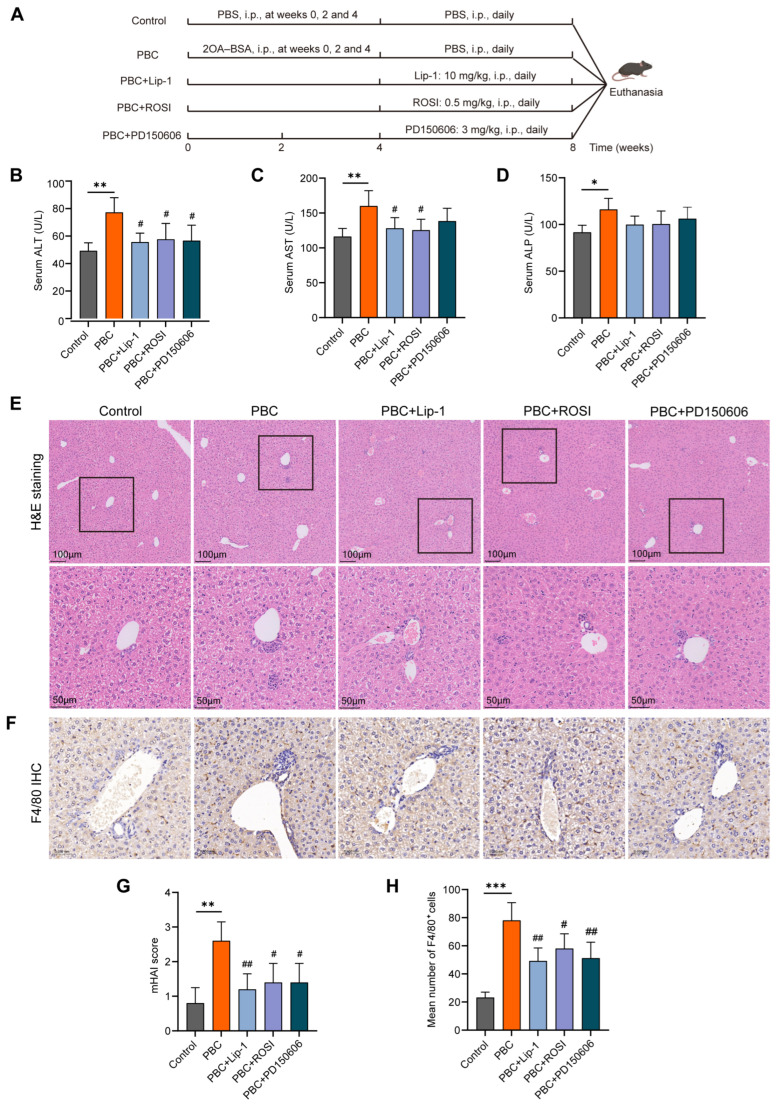
Inhibition of ferroptosis or calpain/ACSL4 signaling mitigates 2OA-BSA-induced PBC-like liver injury. (**A**) Schematic of the 2OA–BSA immunization and treatment schedule with Liproxstatin-1 (Lip-1), rosiglitazone (ROSI) and PD150606. (**B**–**D**) Serum alanine aminotransferase (ALT), aspartate aminotransferase (AST) and alkaline phosphatase (ALP) levels in each group (*n* = 5). (**E**) Representative H&E-stained liver sections (upper panels, low magnification; lower panels, corresponding high-magnification views). (**F**) Representative immunohistochemical staining for F4/80 in liver sections from each group. (**G**) mHAI scores (*n* = 5). (**H**) Quantification of F4/80^+^ cells (number of positive cells per field; *n* = 5). Data are shown as mean ± SD. * *p* < 0.05, ** *p* < 0.01, *** *p* < 0.001 vs. control; # *p* < 0.05, ## *p* < 0.01 vs. PBC.

Given the ferroptosis-associated phenotype observed in hepatic macrophages in 2OA-BSA-induced PBC-like disease, we next tested whether pharmacological inhibition of ferroptosis or blockade of the calpain/ACSL4 axis ameliorates cholangitis in vivo. After disease induction, mice received daily intraperitoneal Lip-1, ROSI or PD150606 for 28 days starting on day 30 (Figure 6A). No treatment-related changes in general condition or body weight were observed during dosing. Relative to controls, PBC mice exhibited significantly elevated serum ALT, AST and ALP (Figure 6B–D). Lip-1 and ROSI significantly reduced ALT and AST, whereas PD150606 reduced ALT and showed a non-significant decrease in AST; none of the three interventions significantly lowered ALP (Figure 6B–D). Histological analysis revealed dense mononuclear inflammatory infiltrates in portal tracts and lobules in PBC mice, which were attenuated by Lip-1, ROSI or PD150606, with corresponding reductions in mHAI scores (Figure 6E,G). Consistently, immunohistochemistry for F4/80 showed marked macrophage accumulation in PBC livers, and all three treatments reduced F4/80^+^ cell abundance in portal and lobular regions (Figure 6F,H). Together, these data indicate that inhibiting ferroptosis or targeting the calpain/ACSL4 axis mitigates biochemical and histological liver injury in this PBC-like model and is accompanied by reduced hepatic macrophage accumulation.

### 3.6. BMDMs from PBC Mice Show Heightened Ferroptotic Susceptibility and Activation of the Calpain/ACSL4 Module

**Figure 7:**
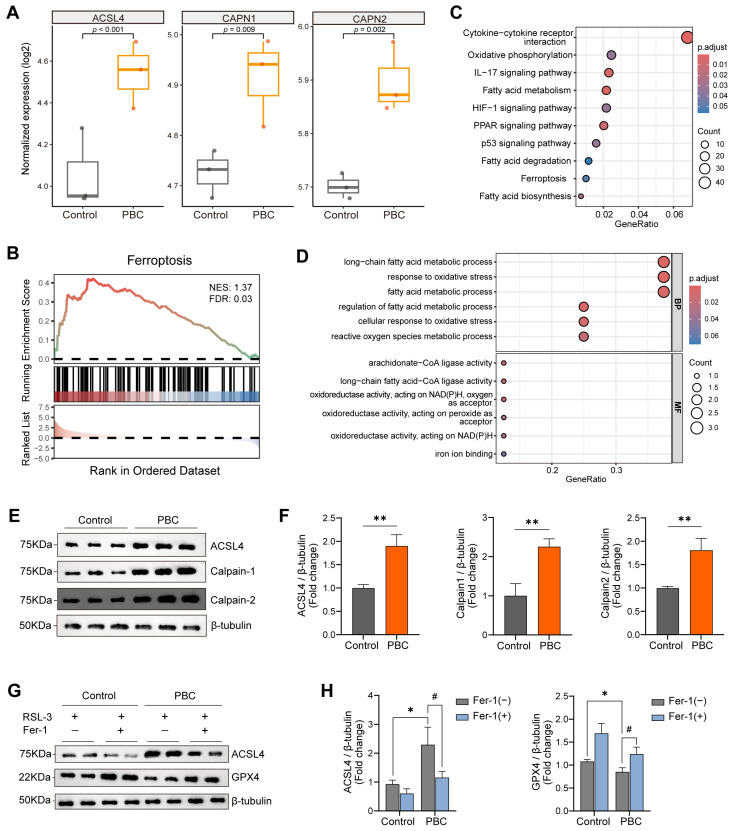
BMDMs from PBC mice show heightened ferroptotic susceptibility and engagement of the calpain/ACSL4 axis. (**A**) Bulk RNA-seq showing transcript levels of ACSL4, CAPN1 and CAPN2 in BMDMs from control and PBC mice. (**B**) GSEA showing significant enrichment of the Ferroptosis gene set in BMDMs from PBC mice. The colored curve represents the running enrichment score, black vertical lines indicate gene hits in the ranked gene list, and the dotted line represents the zero enrichment score. (**C**,**D**) KEGG and Gene Ontology (GO) pathway enrichment bubble plots for differentially expressed genes in BMDMs. (**E**,**F**) Baseline Western blot analysis of ACSL4, calpain-1, and calpain-2 in BMDMs and corresponding densitometric quantification (*n* = 3). (**G**,**H**) Western blot analysis and semiquantitative assessment of ACSL4 and GPX4 in BMDMs treated with RSL3 in the presence or absence of Ferrostatin-1 (Fer-1) (*n* = 3). Data are shown as mean ± SD. * *p* < 0.05, ** *p* < 0.01 vs. control or the indicated comparison group; # *p* < 0.05 vs. the corresponding Fer-1(−) group.

To assess whether macrophages from PBC mice are intrinsically prone to ferroptosis, we generated BMDMs from control and PBC mice and performed bulk RNA sequencing alongside functional assays. GSEA showed significant enrichment of the “Ferroptosis” gene set in BMDMs from PBC mice (Figure 7B). Consistently, KEGG analysis showed that upregulated genes were enriched in pathways linked to ferroptosis, oxidative phosphorylation and fatty acid metabolism (Figure 7C), whereas Gene Ontology analysis highlighted processes involving long-chain fatty acid metabolism, responses to oxidative stress and iron/oxidoreductase-related activities (Figure 7D), suggesting metabolic and redox remodeling in a ferroptosis-prone state. At the transcript level, ACSL4, CAPN1 and CAPN2 were increased in PBC BMDMs compared with controls (Figure 7A). This pattern was recapitulated at baseline protein levels, with higher ACSL4 and calpain-1/2 abundance in PBC-derived BMDMs (Figure 7E,F). Upon RSL3 challenge, PBC BMDMs exhibited more pronounced ferroptosis-associated molecular changes, including further induction of ACSL4 and greater suppression of GPX4. Importantly, Fer-1 markedly reversed these RSL3-induced changes in both groups (Figure 7G,H), supporting heightened, pharmacologically reversible ferroptosis susceptibility in BMDMs from PBC mice.

### 3.7. Calpain/ACSL4 Axis-Associated Ferroptosis in BMDMs Is Pharmacologically Reversible In Vitro

**Figure 8:**
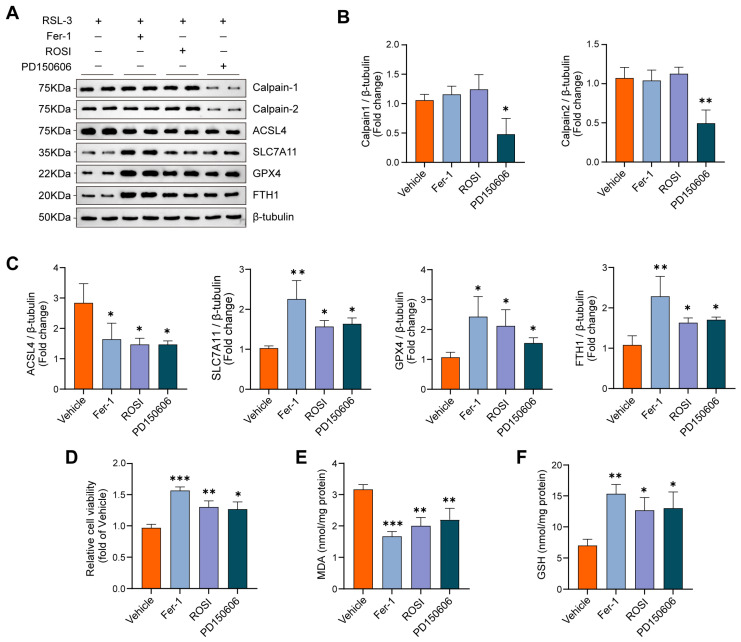
Inhibition of ferroptosis or the calpain/ACSL4 axis attenuates ferroptosis-associated phenotypes in BMDMs from PBC mice. (**A**) Western blot analysis of calpain-1, calpain-2, ACSL4, SLC7A11, GPX4, and FTH1 in BMDMs from PBC mice treated with RSL3 following pretreatment with Ferrostatin-1 (Fer-1), rosiglitazone (ROSI), or PD150606. (**B**,**C**) Densitometric quantification of the indicated proteins (*n* = 3). (**D**) Relative viability of BMDMs assessed by CCK-8 (*n* = 3). (**E**,**F**) Intracellular malondialdehyde (MDA) and glutathione (GSH) levels across treatment groups (*n* = 3). Data are shown as mean ± SD. * *p* < 0.05, ** *p* < 0.01, *** *p* < 0.001.

To further interrogate the calpain/ACSL4 axis in macrophage ferroptosis, we challenged primary BMDMs from PBC mice with RSL3 and assessed pharmacological interventions. Relative to RSL3 alone, pretreatment with Fer-1, ROSI or PD150606 mitigated ferroptosis-associated molecular changes. RSL3 increased ACSL4 abundance and reduced SLC7A11, GPX4 and FTH1, whereas Fer-1, ROSI and PD150606 decreased ACSL4 and restored SLC7A11, GPX4 and FTH1 to varying extents (Figure 8A,C). Notably, PD150606 also reduced CAPN1/2 protein levels, consistent with effective calpain inhibition upstream of the ACSL4-linked program (Figure 8B). Functionally, each intervention improved BMDM viability following RSL3 exposure, lowered intracellular MDA and increased GSH (Figure 8D–F). Together, these data support a regulatory contribution of the calpain/ACSL4 axis to ferroptosis in BMDMs and demonstrate that this phenotype is pharmacologically reversible in vitro.

## 4. Discussion

**Figure 9:**
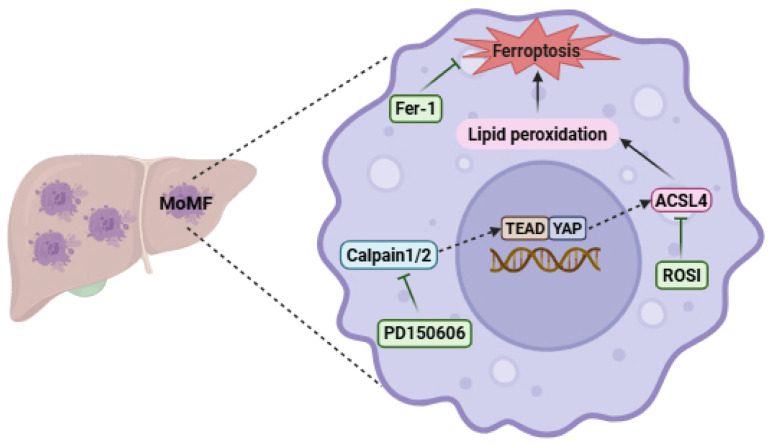
Proposed model linking the calpain/ACSL4 axis to MoMF ferroptosis in PBC and its pharmacological modulation. Dashed arrows indicate putative indirect regulatory links based on previous reports.

In this study, we integrated human single-cell transcriptomics, histological analyses of patient liver tissue, and complementary in vivo and in vitro experiments to delineate a MoMF-centered ferroptotic program associated with cholangitis progression in PBC (Figure 9). PBC livers exhibited increased MoMF infiltration, amplified inflammatory crosstalk between MoMFs and cholangiocytes, and the most pronounced induction of ferroptosis-related activity within MoMFs, with ACSL4 emerging as a key component. Pharmacological inhibition of calpain or ACSL4 attenuated ferroptosis-associated molecular changes and functional readouts in vivo and in isolated macrophages, and was accompanied by reduced cholangitis-related liver injury. Together, these convergent data implicate MoMFs as a major cellular compartment in which ferroptosis is engaged in PBC and support the calpain/ACSL4 axis as a therapeutically tractable target.

In PBC, an autoimmune cholangitis, MoMFs are thought to play a central role in sustaining chronic inflammation and tissue remodeling [8,10,11]. Consistent with this view, our single-cell RNA-seq analysis revealed a marked expansion of MoMFs in PBC livers, accompanied by strengthened inflammatory crosstalk with cholangiocytes and the most pronounced induction of ferroptosis-related transcriptional programs within MoMFs. Triple immunofluorescence staining of infiltrating macrophages and pseudotime analysis provided concordant support, showing increased ACSL4/4-HNE-associated signals and rising ferroptosis module scores along the MoMF continuum in PBC. Together, these observations suggest that heightened ferroptotic susceptibility in MoMFs may contribute to the maintenance and amplification of cholangitis-associated inflammation. Although resident Kupffer cells and other hepatic lineages also showed increased FerrScore, MoMFs displayed the largest shift together with increased representation and strengthened cholangiocyte crosstalk, supporting their prioritization for downstream analyses. Among regulated cell death pathways involved in hepatic inflammation, ferroptosis-related signatures ranked among the most significantly enriched programs in our transcriptomic analyses. On this basis, we focused subsequent mechanistic experiments on ferroptosis-associated changes in MoMFs.

Ferroptosis is a regulated, non-apoptotic form of cell death driven by iron-dependent lipid peroxidation and has attracted increasing attention in recent years. Macrophage ferroptosis has been implicated in tissue injury and inflammatory amplification across diverse chronic disease settings, including cancer, atherosclerosis and autoimmune disorders [37,38,39]. Mechanistically, ferroptotic macrophages have been reported to release pro-inflammatory cytokines and chemokines (including IL-1β, IL-6, TNF-α and CCL2), danger-associated molecular patterns such as HMGB1, and labile Fe^2+^, thereby promoting innate immune activation and propagation of inflammation [16,40]. In keeping with these observations, pharmacologic or genetic inhibition of macrophage ferroptosis has shown therapeutic potential in models of atherosclerosis [41] and selected autoimmune diseases [42,43,44] and has been reported to attenuate hepatic inflammation and tissue injury [45,46]. Against this backdrop, we observed the most pronounced induction of ferroptosis-related programs in MoMFs in PBC by scRNA-seq, with tissue-level validation in infiltrating macrophages. Moreover, inhibiting ferroptosis in vivo and in vitro attenuated ferroptosis-associated molecular changes and improved functional readouts, in parallel with reduced inflammatory injury. Given that systematic, cell-type-resolved evidence for ferroptosis in PBC remains limited, our findings provide support for ferroptosis engagement in disease-relevant macrophage compartments and highlight candidate regulatory nodes for therapeutic intervention.

ACSL4, a key enzyme that shapes the pool of polyunsaturated fatty acyl substrates and the susceptibility of membrane lipids to peroxidation, has emerged as a critical regulator of ferroptosis in several nonmalignant disease models, including hepatic ischemia–reperfusion injury [47,48], chronic kidney disease [49,50], inflammatory bowel disease [51,52], Sjögren’s syndrome [53], and systemic lupus erythematosus [54]. Consistently, pharmacological targeting of ACSL4 to restrain ferroptosis has been reported to ameliorate hepatic inflammation and fibrosis [55,56,57]. ROSI has been used in experimental settings to suppress ACSL4-associated, ferroptosis-related lipid remodeling and thereby attenuate ferroptotic injury [58,59]. However, because ROSI is also a classical PPARγ agonist, its protective effects may additionally involve PPARγ-mediated anti-inflammatory regulation in macrophages and biliary injury, in addition to ACSL4-associated ferroptotic lipid remodeling [60,61]. In line with these observations, we found that MoMFs from patients with PBC and from 2OA-BSA-immunized mice displayed an ACSL4-focused program consistent with increased ferroptosis susceptibility. In vivo and in vitro, treatment with ROSI, alongside other ferroptosis-inhibition strategies, partially or markedly reversed ferroptosis-related molecular signatures and was accompanied by improvements in functional measures and attenuation of cholangitis-related phenotypes. These findings support a key role for ACSL4 in shaping ferroptotic vulnerability in MoMFs in PBC and provide a rationale for exploring ACSL4-targeted interventions as a potential strategy to modulate immune-mediated cholangitis.

Calpains are Ca^2+^-dependent cysteine proteases that participate in cellular stress responses and have been implicated as upstream modulators of lipid peroxidation and ferroptotic susceptibility [62,63]. The best-characterized isoforms, calpain-1 and calpain-2, form heterodimeric complexes composed of a large catalytic subunit encoded by CAPN1 or CAPN2 and a common small regulatory subunit encoded by CAPNS1 [64]. In inflammatory macrophage models, genetic inhibition of CAPN1/2 reduces ACSL4 expression and attenuates ferroptosis-associated phenotypes, whereas CAPN1/2 overexpression increases ACSL4 abundance and ferroptosis-related readouts; these effects can be counteracted by ROSI, supporting functional coupling between calpain activity and ACSL4-dependent lipid remodeling [18,65]. Mechanistic work further suggests that calpain-1 can suppress AMPK phosphorylation and promote YAP nuclear translocation and TEAD binding, thereby increasing ACSL4 protein levels and amplifying lipid peroxidation and ferroptosis [19]. In vivo, systemic calpain inhibition reduces tissue calpain activity and lowers ACSL4 levels, accompanied by improvement in skin and lung inflammation and fibrosis, consistent with anti-inflammatory and anti-fibrotic effects of blocking the calpain/ACSL4 axis [18,19]. Importantly, our data do not establish ACSL4 as a direct proteolytic substrate of calpain; rather, the PD150606-associated reduction in ACSL4 should be interpreted as an indirect regulatory effect, potentially consistent with the previously reported AMPK–YAP/TEAD mechanism. In addition, inhibition of calpain-1 has been reported to attenuate hepatic inflammation and improve liver function indices, supporting the feasibility of targeting calpain signaling in liver disease [66,67]. Against this backdrop, our data support engagement of a calpain/ACSL4 axis in PBC-associated macrophages. In MoMFs from the human scRNA-seq dataset, CAPN1/2 exhibited higher detection frequencies in PBC than in controls, whereas mean expression per cell remained modest. This pattern, characterized by greater prevalence with limited per-cell upregulation, is consistent with the predominant post-translational regulation of calpains via Ca^2+^ availability and CAPNS1-dependent complex stability [64,68], and suggests that a larger fraction of MoMFs is poised to mount calpain-mediated responses. Along the MoMF pseudotime continuum, CAPN1/2 and ACSL4 increased in parallel, mirroring the late rise in ferroptosis module scores. In the PBC mouse model, CAPN1/2 differences were more pronounced in BMDMs, and the calpain inhibitor PD150606 attenuated RSL3-induced ferroptosis in vitro, in parallel with improvements in cholangitis-associated measures in vivo and complementing the effects of ROSI and Fer-1. Collectively, evidence from single-cell profiling, supportive histological evidence and in vivo/in vitro perturbation implicates a drug-responsive calpain/ACSL4 module that modulates ferroptotic susceptibility in MoMFs in PBC and supports further evaluation of this axis as a translational target.

Our study has several limitations. First, we used a 2OA-BSA-induced mouse model that recapitulates key features of cholangitis but cannot fully capture the clinical and biological heterogeneity of human PBC; extrapolation to patients should therefore be made with caution. In addition, only female mice were used in this study, consistent with the female predominance of PBC and commonly used murine PBC models. However, the estrous cycle was not monitored, and the potential effects of hormonal fluctuations on immune and inflammatory responses cannot be fully excluded. Second, the human liver mIF analysis was performed in a limited number of samples and should be interpreted as supportive tissue-level evidence rather than definitive clinical validation; larger PBC cohorts will be required to confirm the generalizability of these findings. Third, although prior genetic studies support calpain-dependent regulation of ACSL4 and macrophage ferroptosis, our evidence in primary BMDMs from PBC mice is largely pharmacological, based on the reversibility of multiple molecular and functional readouts. We did not directly measure calpain activity or perform siRNA/CRISPR-mediated knockdown of CAPN1/2 or ACSL4 in BMDMs; therefore, the causal specificity of the calpain/ACSL4 axis requires further genetic validation. In addition, some in vitro assays were performed with a limited number of independent biological replicates, which reduces statistical power and warrants confirmation in larger independent experiments. Finally, although ferroptosis-related alterations were most prominent in MoMFs, cholangiocytes also displayed increased ferroptosis-related signatures, raising the possibility that PD150606, ROSI and Lip-1 act across multiple cellular compartments. The relative contribution of cholangiocytes to ferroptosis-associated pathology in PBC remains to be defined.

## 5. Conclusions

In conclusion, integrating omics-based analyses with complementary experimental models, we identify a ferroptosis program in PBC that is most prominently engaged in MoMFs and is centered on ACSL4. Across both the 2OA-BSA-induced mouse model and primary BMDM systems, inhibiting ferroptosis or pharmacologically targeting calpain or ACSL4 attenuated ferroptosis-associated molecular changes and functional deficits, in parallel with improved cholangitis-related liver injury. Together, these data implicate the calpain/ACSL4 axis as a drug-responsive regulatory node that may shape ferroptotic susceptibility in MoMFs and contribute to inflammatory pathology in PBC, providing a rationale for further evaluation of this pathway as a therapeutic strategy.

## Figures and Tables

**Table 1:** Drugs and chemicals.

Chemicals	Vendor	Catalog No.	Country
Complete Freund’s adjuvant	Sigma-Aldrich	F5881	USA
Incomplete Freund’s adjuvant	Sigma-Aldrich	F5506	USA
Liproxstatin-1	AbMole	M8531	USA
Rosiglitazone	AbMole	M1894	USA
PD150606	AbMole	M7764	USA
RSL3	AbMole	M9060	USA
Ferrostatin-1	AbMole	M2698	USA
Macrophage colony-stimulating factor	MCE	HY-P7085	USA

## Data Availability

Publicly available datasets analyzed in this study are available in the Gene Expression Omnibus (GEO) database under accession number [GSE243981]. Other data supporting the findings of this study are available from the corresponding author upon reasonable request.

## Supplementary Materials

The following supporting information can be downloaded at: https://www.mdpi.com/article/10.3390/biomedicines14061208/s1, Figure S1: Ferroptosis-related transcriptional changes and calpain gene associations in MoMFs from human PBC livers.

## Abbreviations

The following abbreviations are used in this manuscript:

PBC	Primary biliary cholangitis
MoMF	Monocyte-derived macrophage
scRNA-seq	Single-cell RNA sequencing
DC	Dendritic cells
T/NK	T cells/natural killer cells
GSEA	Gene set enrichment analysis
KEGG	Kyoto Encyclopedia of Genes and Genomes
GO	Gene Ontology
ACSL4	Acyl-CoA synthetase long-chain family member 4
4-HNE	4-hydroxynonenal
MDA	Malondialdehyde
GSH	Glutathione
Lip-1	Liproxstatin-1
Fer-1	Ferrostatin-1
ROSI	Rosiglitazone
RSL3	RAS-selective lethal 3
2OA–BSA	2-octynoic acid–bovine serum albumin conjugate
AMA-M2	Antimitochondrial antibody M2
ALT	Alanine aminotransferase
AST	Aspartate aminotransferase
ALP	Alkaline phosphatase
