# Supplementary material for: Monocyte-Derived Macrophage Ferroptosis Amplifies Cholangitis in Primary Biliary Cholangitis via a Calpain/ACSL4 Axis
Source: Biomedicines. 2026 May 27;14(6):1208. doi: 10.3390/biomedicines14061208 (PMC13296124; doi:10.3390/biomedicines14061208)

Figure S1. Ferroptosis-related transcriptional changes and calpain gene associations in MoMFs from human PBC livers.

(A) Heatmap of ferroptosis-related genes in MoMFs comparing control and PBC livers. (B) Dot plot showing CAPN1, CAPN2 and CAPNS1 expression in MoMFs by group; dot size indicates the percentage of cells expressing each gene and color denotes average expression. (C) Spearman correlation matrix between ferroptosis module score (FerrScore) and expression of ACSL4, CAPN1, CAPN2 and CAPNS1 in MoMFs; numbers indicate correlation coefficients. Statistical significance is denoted as *P < 0.05, **P < 0.01, ***P < 0.001.

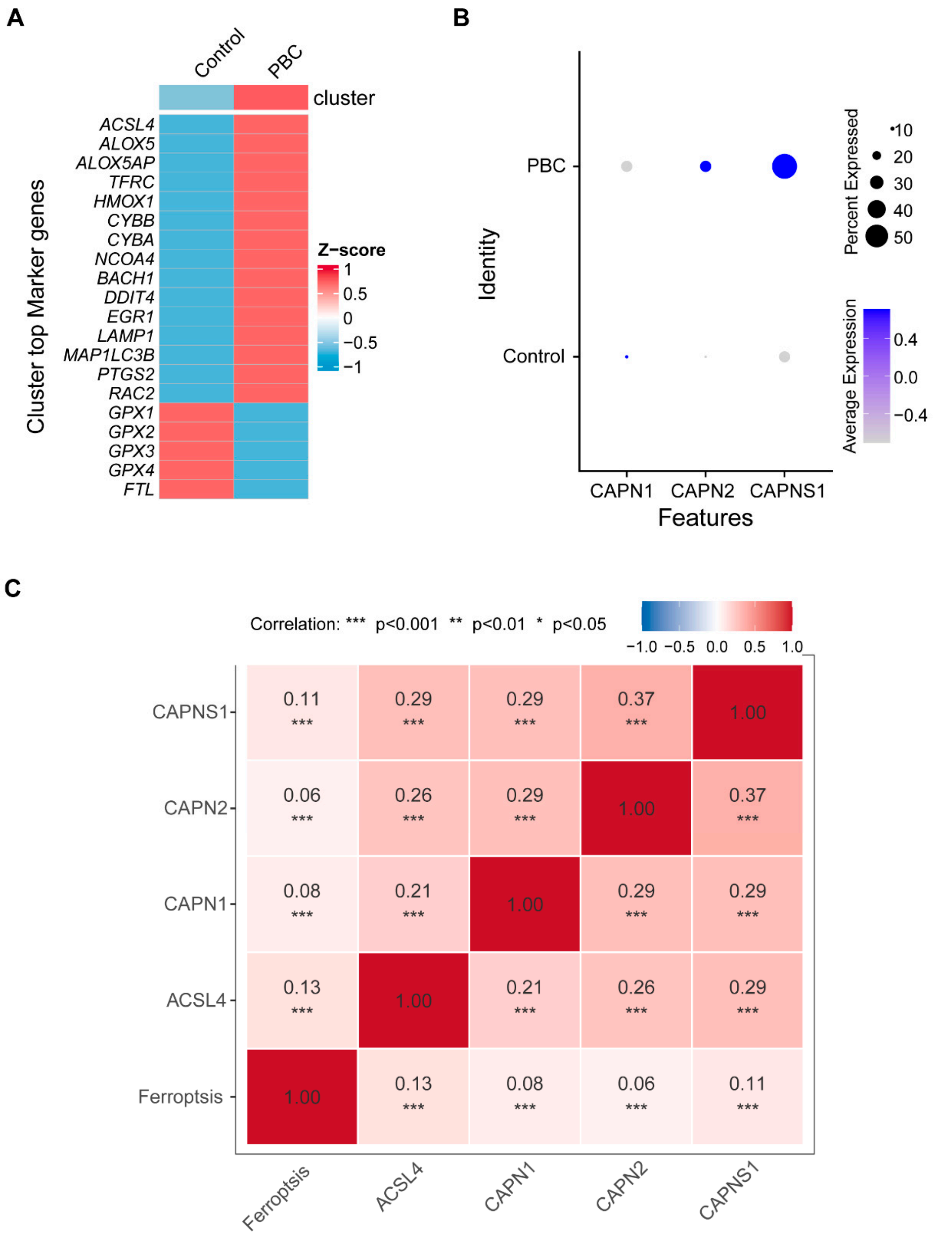

Supplement: Supplementary file 1 [file biomedicines-14-01208-s001.zip › biomedicines-4289295-supplementary.pdf]
